# Worst-Case X-ray Photon Energies in Ultrashort Pulse Laser Processing

**DOI:** 10.3390/ma15248996

**Published:** 2022-12-16

**Authors:** Katrin Böttcher, Mayka Schmitt Rahner, Ulf Stolzenberg, Sebastian Kraft, Jörn Bonse, Carsten Feist, Daniel Albrecht, Björn Pullner, Jörg Krüger

**Affiliations:** 1 Bundesanstalt für Materialforschung und -prüfung (BAM), Unter den Eichen 87, 12205 Berlin, Germany; 2 Physikalisch-Technische Bundesanstalt (PTB), Bundesallee 100, 38116 Braunschweig, Germany

**Keywords:** ultrashort pulsed laser, X-ray emission, X-ray spectrum, X-ray energies, X-ray dose rate, radiation protection

## Abstract

Ultrashort pulse laser processing can result in the secondary generation of unwanted X-rays if a critical laser irradiance of about 10^13^ W cm^−2^ is exceeded. Spectral X-ray emissions were investigated during the processing of tungsten and steel using three complementary spectrometers (based on CdTe and silicon drift detectors) simultaneously for the identification of a worst-case spectral scenario. Therefore, maximum X-ray photon energies were determined, and corresponding dose equivalent rates were calculated. An ultrashort pulse laser workstation with a pulse duration of 274 fs, a center wavelength of 1030 nm, pulse repetition rates between 50 kHz and 200 kHz, and a Gaussian laser beam focused to a spot diameter of 33 μm was employed in a single pulse and burst laser operation mode. Different combinations of laser pulse energy and repetition rate were utilized, keeping the average laser power constant close to the maximum power of 20 W. Peak irradiances *I*_0_ ranging from 7.3 × 10^13^ W cm^−2^ up to 3.0 × 10^14^ W cm^−2^ were used. The X-ray dose equivalent rate increases for lower repetition rates and higher pulse energy if a constant average power is used. Laser processing with burst mode significantly increases the dose rates and the X-ray photon energies. A maximum X-ray photon energy of about 40 keV was observed for burst mode processing of tungsten with a repetition rate of 50 kHz and a peak irradiance of 3 × 10^14^ W cm^−2^.

## 1. Introduction

Already since the 1980s, it has been established that X-rays can be emitted from laser-induced plasmas. Early investigations were performed in vacuum [1]. In the early 2000s, the first investigations during ultrashort pulse laser materials processing of copper with a repetition rate of 1 kHz in air were conducted. Thogersen et al. [2] measured a maximum dose rate of X-rays of approximately 10 mSv h^−1^ at a distance of 13 cm and Bunte et al. [3] determined a maximum dose rate of approximately 50 mSv h^−1^ at a distance of 10 cm, whereby the specifically measured radiological operational quantity was not explicitly specified in both studies. For steel and tungsten processed at higher repetition rates of 400 kHz, Legall et al. [4] found in 2018 even higher dose equivalent rates *Ḣ*′(0.07) of 163 mSv h^−1^ at a distance of 42 cm exceeding typical radiation protection safety limits, which revealed a potential safety risk for the operating staff of the ultrashort pulse laser (USPL) machine.

Several investigations were performed to analyze the X-ray emission for different laser and process parameters during ultrashort pulse laser machining of various technical materials [5,6,7,8,9,10,11,12,13,14,15,16,17]. Recently, possibly harmful X-ray emission was observed already at laser irradiances below 10^13^ W cm^−2^ [18,19]. Especially the burst modes of laser machines were identified as a configuration that can lead to very high dose rates in the ultrashort laser pulse processing of metals [20,21]. During burst mode processing, pulse trains (bursts) with up to 100 sub-pulses per burst and intra-burst pulse repetition rates in the MHz to GHz range are typically generated by current commercial laser systems [22,23]. When using burst mode settings, the pulse energy from a single laser pulse is divided into a predefined number of sub-pulses with corresponding (lower) sub-pulse energies.

Besides the dose equivalent rates, the spectral flux of the emitted X-ray photons is of special interest as the transmission of X-rays through a radiation protection housing strongly depends on its spectral distribution.

This study aims to identify an X-ray photon spectrum showing the maximum X-ray energies that can be generated during ultrashort pulse laser material processing of steel and tungsten in air, the so-called “worst-case spectrum”. For that, the X-ray spectra were measured during the machining process for different laser and process parameters. The corresponding dose equivalent rates were calculated from the spectra.

## 2. Materials and Methods

### 2.1. Ultrashort Pulse Laser System

The investigation was performed with an ultrashort pulse laser machine emitting an average power of 20 W at a center wavelength of 1030 nm (GL.evo, GFH GmbH, Deggendorf, Germany), equipped with a Pharos-Laser (PH2-20W, Light Conversion, Vilnius, Lithuania). The laser pulse duration of 274 fs was kept constant. The laser beam was focused on the workpiece featuring an 1/e^2^-spot diameter of 33 μm. This value was taken from a configuration file provided by the manufacturer of the laser machine. It was operated here in an industrial setting that regularly does not involve an individual beam diameter evaluation for each laser process. The detailed processing parameters are listed in Table 1. Different combinations of laser pulse energy and repetition rate were chosen, whereby the laser pulse energy was varied between 91 µJ and 371 μJ and the pulse repetition rate between 50 kHz and 200 kHz. For each combination of laser pulse energy and repetition rate, single pulse and burst mode experiments were performed.

For the burst mode investigations, the single pulse (with 100% energy) is split into two sub-pulses showing equal energy of 50% of the total single pulse energy. The two sub-pulses have a temporal separation of 200 ps here.

Laser peak irradiances *I*_0_ in a range of 7.3 × 10^13^ W cm^−2^ to 3.0 × 10^14^ W cm^−2^ were calculated under the assumption of a temporal and spatial Gaussian distribution of the laser pulse and an angle of incidence of 0°

(1) $$I_{0}=8\sqrt{\frac{4\ln2}{\pi }}\frac{Q}{\tau_{H}\cdot \pi \cdot d^{2}},$$

where *Q* is the pulse energy, *τ_H_* is the pulse duration and *d* is the laser beam spot diameter (1/e^2^) [24].

The calculation of the irradiance for the burst mode in Table 1 considers the total energy of the burst (which equals the single pulse energy). Three different repetition rates (50 kHz, 100 kHz, and 200 kHz) and pulse energies (371 μJ, 184 μJ, and 91 µJ) were selected, resulting in an average power of 18.2 W to 18.6 W close to the maximum average power of the laser of 20 W. The experiments were performed with flat 5 mm thick plates of tungsten (99.97% purity) and steel alloy (St37) targets. The target surfaces were prepared by parallel surface grinding achieving an average roughness of 1.6 µm to 4 µm. With a laser beam scanning configuration, 1.5 × 1.5 cm^2^ squares were treated. The laser processing time was 250 s for each square.

### 2.2. Spectral X-ray Emission

The measurement of the spectral X-ray emission in the context of ultrashort pulse laser processing is challenging due to non-matching energy detection ranges of different spectrometers, electromagnetic compatibility (EMC) requirements, possible pile-up effects, and potential X-ray screening through the environment. Up to now, X-ray energies between approximately 2 keV and 30 keV were reported during ultrashort pulse laser processing in air [4]. However, detectors based on different sensor materials measure reliably in different X-ray energy ranges. Typical silicon drift detectors (SDDs) are only applicable for photon energies up to approximately 15 keV as the quantum efficiency of 500 µm thick silicon rapidly drops for larger energies (see for example Figure 1 in [25]). Strüder et al. reported a quantum efficiency that was always above 85% for photon energies between 500 eV and 11 keV for a 450 μm thick SDD [26]. However, as most of the X-ray spectra measured during laser material interactions cover a rather low photon energy range, measurements with an SDD can still provide valuable insights. As an alternative, CdTe detectors exhibit a much larger energy detection range than SDDs. For the X-123 spectrometer (Amptek Inc., Bedford, MA, USA) with a sensor thickness of 1000 µm for example, the manufacturer specifies an optimum energy range from 5 keV to 100 keV. The manufacturer data give a minimum intrinsic efficiency of 98% for photon energies between 10 keV and 50 keV.

For CdTe spectrometers, previous measurements indicate that the spectra can be influenced by noise and electronical issues caused by electromagnetic fields emitted from the plasma [14]. Unwanted electronic events may happen because of the laser-target interactions that create strong electromagnetic pulses potentially inducing electronic noise in the data acquisition system [27]. Additionally, the spectra for both detector types can be affected by pile-up [4].

**Figure 1:**
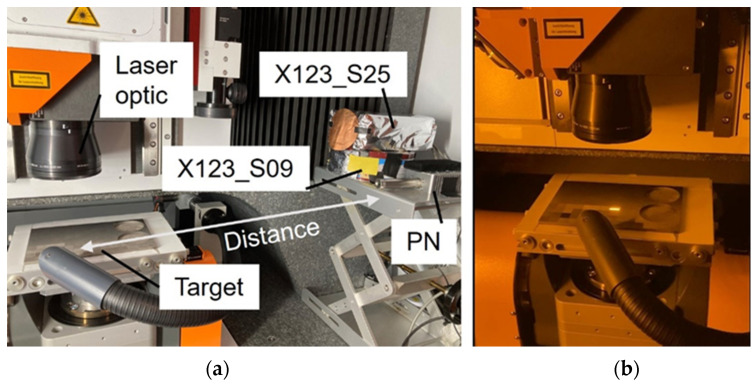
Experimental setup, (**a**) Arrangement of the spectrometers, (**b**) Image during laser processing.

For the reliable measurement of X-ray spectra and to compensate for some disadvantages of the individual spectrometer types, a comparative study using three spectrometers simultaneously was conducted in this work. The spectrometers were placed 60 cm to 70 cm away from the laser interaction point within the housing of the industrial ultrashort pulse laser machining system. The experimental setup with the arrangement of the detectors can be seen in Figure 1a,b showing a photograph of the machining process with the optics, the target (with the bright optical plasma emission) and the exhaust system. The measurements were performed in ambient air.

For the measurements of the higher X-ray photon energies (> 13 keV), two CdTe-based spectrometers (Amptek X-123) were used. The first X-123 spectrometer had a sensor size of 3 × 3 × 1 mm^3^ (X123_S09) and the second had a sensor size of 5 × 5 × 1 mm^3^ (X123_S25). Both X-123 spectrometers were equipped with a 100 µm Beryllium window. For the lower energy range (< 13 keV), an SDD-based spectrometer (PNDetector with the designation “XRS-30-128-100-BeP Complete” with module type SDD-30-128-BeP) with a sensor area of 30 mm^2^, a thickness of 450 ± 20 µm and a beryllium window with a thickness of 8 µm was used. This spectrometer is referred to as “PN” in the following.

The X-ray emission investigations showed EMC issues for both CdTe spectrometers (X-123). The SDD (PN) was not affected. To reduce the influence due to the EMC issues, the X-123 spectrometers were encased in special housings. The X123_S09 was placed in a tinplate box and the X123_S25 in an aluminum box. To further suppress electronic noise, aluminum foils were put in front of the measurement window. The thickness of the aluminum foils (different thicknesses for X123_S09 and X123_S25) was calculated from absorption measurements using an X-ray tube with and without aluminum foils (see Section 3.1). To evaluate the influence of pile-up effects on the measured spectra, the measurements were performed with and without a 50 µm thick copper foil (see Section 3.1).

**Figure 2:**
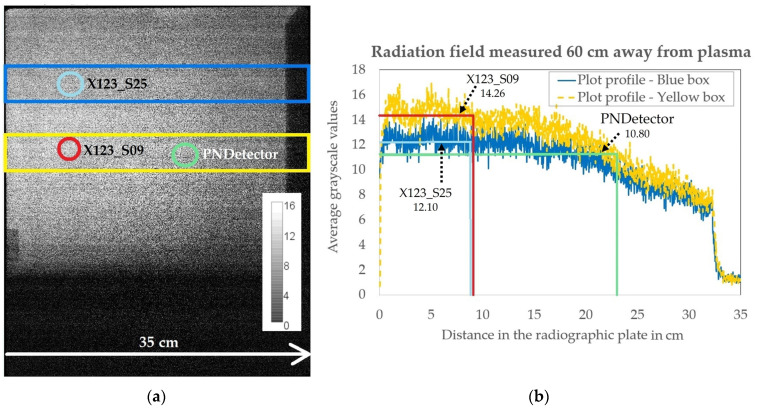
Grayscale image captured with the radiographic imaging plate (**a**) placed 10 cm in front of the three spectrometers used in this study. The blue and yellow rectangles are the areas used to calculate the X-ray intensity profile shown in the plot (**b**). The continuous decrease in grayscale value with an increased distance on the *x*-axis revealed that the PN spectrometer (PNDetector) received less X-ray intensity in comparison to the two X-123 spectrometers. Additionally, X123_S25 received about 14.5% less radiation than the X123_S09 spectrometer.

The placement of the spectrometers with respect to the X-ray emission source and its spatial homogeneity was controlled with a radiographic imaging plate placed at a distance of 60 cm from the laser-induced emission source about 10 cm in front of the three spectrometers. A 2-D Radiography Scanner CR 35 NDT Plus system (DÜRR, Stuttgart, Germany) was used. Figure 2a shows the grayscale image of the X-ray field recorded by the 2-D radiography plate along with the positions of the detectors that are marked by green, red, and blue circles, respectively. To compare the X-ray intensities arriving at the three spectrometers, the recorded data contained in the blue and yellow boxes were then binned vertically and then plotted as a function of the horizontal position in Figure 2b as blue and yellow curves. The additional green, red, and blue vertical lines mark the individual positions of the three spectrometers, while the corresponding horizontal lines indicate the average grayscale level as a measure of the local X-ray intensity incident to the detector. The spectrometer that received more intensity of laser-generated X-ray intensity (a higher average grayscale value) was the X123_S09. The X123_S25 and the PN received in comparison to X123_S09 about 14.5% and 24% less X-ray intensity during the measurements.

## 3. Results

### 3.1. Spectrometer Characterization

**Figure 3:**
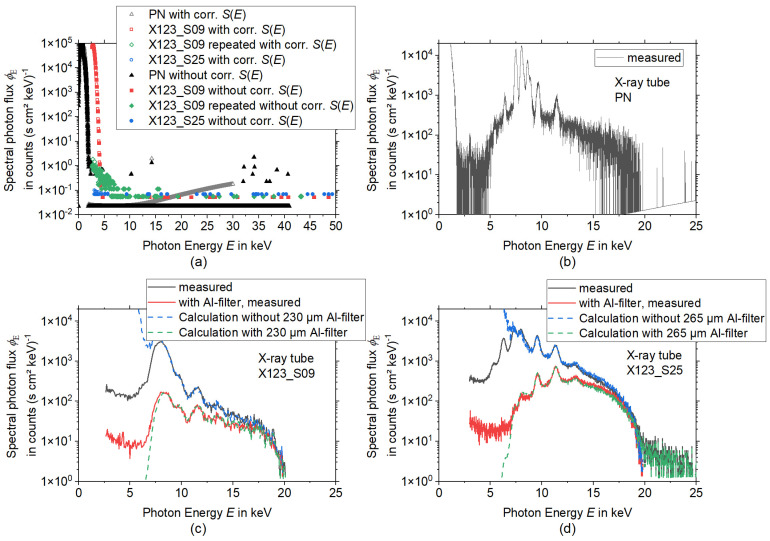
Characterization of the spectrometers without laser material processing, (**a**) Background measured inside the running USPL machine without laser processing (with and without correction of the spectral sensitivity of the sensor *S*(*E*)), (**b**) X-ray tube spectrum measurement with the PN spectrometer, (**c**) X-ray tube spectra measured with X123_S09 with and without Al-filter (solid lines) along with two calculated spectra (dashed curves), (**d**) X-ray tube spectra measured with X123_S25 with and without Al-filter (solid lines) along with two calculated spectra (dashed curves). Note the logarithmic scaling of the ordinate axes.

The spectrometers were characterized by background measurements (Figure 3a) and measurements of an X-ray tube (Figure 3b–d). For a comparison between the different spectrometer properties, the same (known) spectrum of an X-ray tube (Amptek Mini-X Gold (Au) X-ray tube) operated at a current of 10 mA and a voltage of 20 kV was measured by all three devices.

Every data point in the measured pulse height spectrum *X*(*E*) is corrected with the spectral sensitivity of the sensor *S*(*E*) and normalized to the area and time with the sensor area *A* and measuring time interval *t* and yields the photon flux *Φ*_0_(*E*):

(2) $$\Phi_{0}\left(E\right)=\frac{X\left(E\right)}{A\;t\;S\left(E\right)}$$

Typically, in order to determine the photon flux from the pulse height spectrum, an unfolding procedure is required which depends on the inverse response matrix of the detector. Here, due to the low energies and negligible influences due to scattering because of the very low energies, the detector response was estimated by employing only the sensitivity of the detector. Afterward, the spectral photon flux *Φ_E_*(*E*) was calculated by the normalization of *Φ*_0_ with the energy channel’s width ∆*E*

(3) $$\Phi_{E}\left(E\right)=\frac{\Phi_{0}\left(E\right)}{∆E}$$

To have a comparative situation like during the measurement of the X-ray emission from the laser generated plasma, the X-ray tube was positioned inside the USPL machine on the position, where the laser-induced plasma generation would take place. The X-ray tube measurements were performed when the USPL machine was turned off.

Furthermore, the background signal for all detectors was measured during the USPL machine was running, but no laser material processing took place (Figure 3a). This way, it was proven, whether the detection was disturbed by any influence from the USPL machine such as electronic issues, for example, but without the additional influence from the transient plasma generation during the laser processing.

From Figure 3a,b one can see that the PN spectrometer has always a high-intensity count range in the low-energy region up to around 2 keV. This noise signal happens due to the high sensitivity of the silicon detector material. This signal also occurred during the background measurement and is independent of the X-ray tube, see Figure 3a.

The PN spectrometer shows the highest spectral resolution of the characteristic peaks from the spectra shown in Figure 3b and the X123_S25 has a higher resolution than the X123_S09, which can be due to the larger sensor size and the higher number of channels of the X123_S25.

For the PN spectrometer signal (Figure 3a) only background noise in the very low count range within the energy region from 2 keV to about 40 keV can be seen. For both Amptek X-123 spectrometers, for energies above 5 keV to 8 keV only background noise with a spectral photon flux *Φ_E_* < 0.1 counts (s cm^2^ keV)^−1^ occurs. For the X123_S09 a region with somewhat higher spectral photon flux is found below 8 keV. Repeated measurements showed that this is not a stable signal, and it was not observed during the measurement of the X-ray tube, revealing that the signal is caused by EMC or other electrical issues.

Since all spectral detectors show no significant or stable background within the interesting energy region above 5 keV, a subtraction of the background was not performed. To calculate the influence of the Al foils, which are highly recommended to be used in front of the detector window of an Amptek X-123 spectrometer to avoid EMC issues and spectral artifacts due to pile-up, measurements of the X-ray tube spectrum were performed with and without the aluminum foil. Then the expected spectrum after the transition of aluminum filters with different thicknesses was calculated and the filter thickness was determined with a least-squares-fit. The results can be found in Figure 3c,d.

The spectral calculation is based on linear absorption according to Beer’s law. Hereby, the measured X-ray flux without filters is reduced by *i*-filters with their attenuation coefficient from the NIST database [28] *µ_i_* and thickness *d_i_*.

(4) $$\Phi \left(E\right)=\Phi_{0}\left(E\right)\;e^{-\underset{i}{\sum }\mu_{i}d_{i}}$$

The procedure was repeated in reverse with the measured spectrum with aluminum foils to calculate the spectrum without foils. For the X123_S09 an aluminum thickness of 230 µm (Figure 3c) and for the X123_S25 (Figure 3d) an aluminum thickness of 265 µm was determined. For energies higher than 7 keV the calculation and the measurements are in good agreement. The deviations for energies lower than 7 keV are due to absorption in the beryllium window in front of the CdTe sensor volume and electronic noise for the low photon energies.

### 3.2. Spectroscopy during Ultrashort Pulse Laser Processing

To evaluate the influence of pile-up effects or EMC on the spectra, the measurements were performed during the laser processing with and without a 50 µm thick copper foil. From the measurements without copper foil, the spectra after the transmission through the copper foil were calculated. This way, a comparison between the measurements with copper foil and the calculations could be performed. Deviations indicate that the spectra measured without copper foil are still influenced either by pile-up (in high-energy regions) or by electronic noise induced in the acquisition system of the spectrometers due to low-frequency electromagnetic fields emitted from the laser-induced plasma.

**Figure 4:**
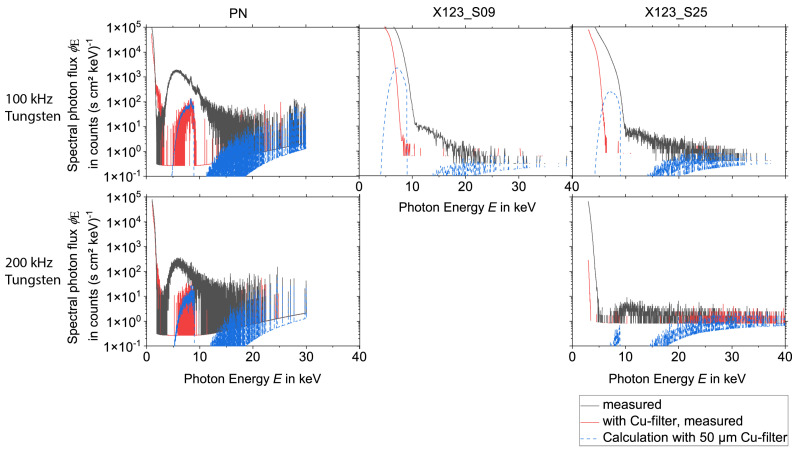
X-ray spectra measured during the laser processing of tungsten without burst mode for the three spectrometers PN, X123_S09, and X123_S25, the measurement with and without Cu-filter was compared with the calculation with copper filter of 50 µm thickness. Note the logarithmic scaling of the ordinate axes.

**Figure 5:**
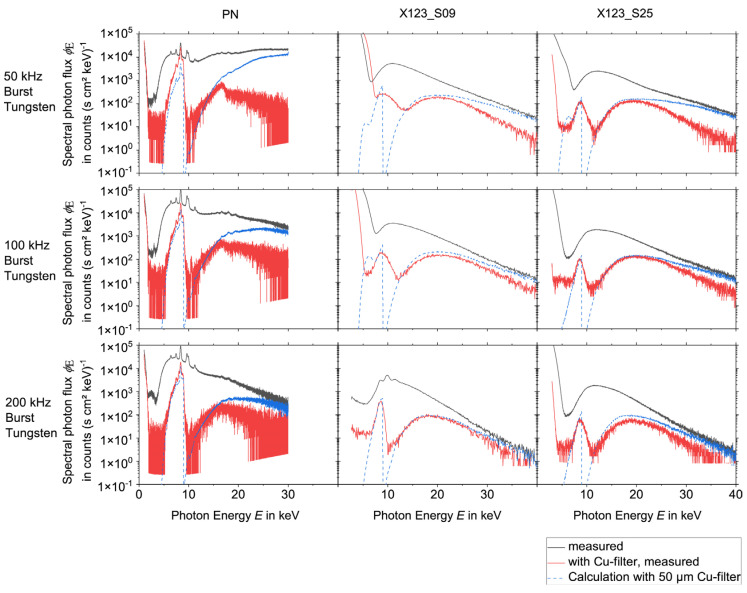
X-ray spectra measured during the laser processing of tungsten with burst mode for the three spectrometers PN, X123_S09, and X123_S25, the measurement with and without Cu-filter was compared with the calculation with copper filter of 50 µm thickness. Note the logarithmic scaling of the ordinate axes.

**Figure 6:**
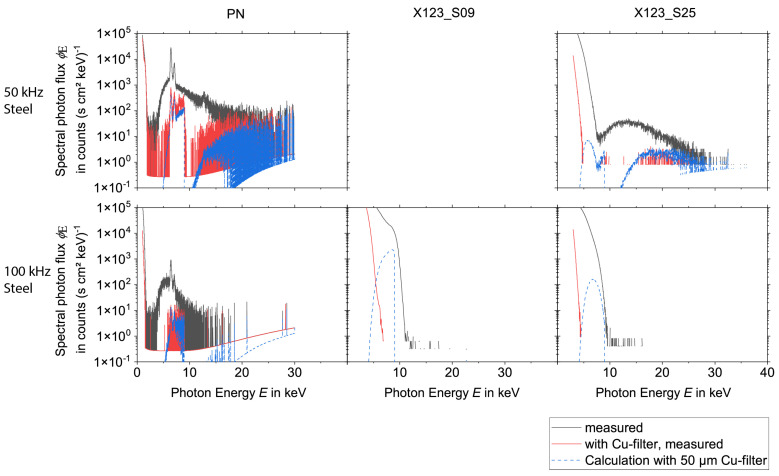
X-ray spectra measured during the laser processing of steel without burst mode for the three spectrometers PN, X123_S09, and X123_S25, the measurement with and without Cu-filter was compared with the calculation with copper filter of 50 µm thickness. Note the logarithmic scaling of the ordinate axes.

**Figure 7:**
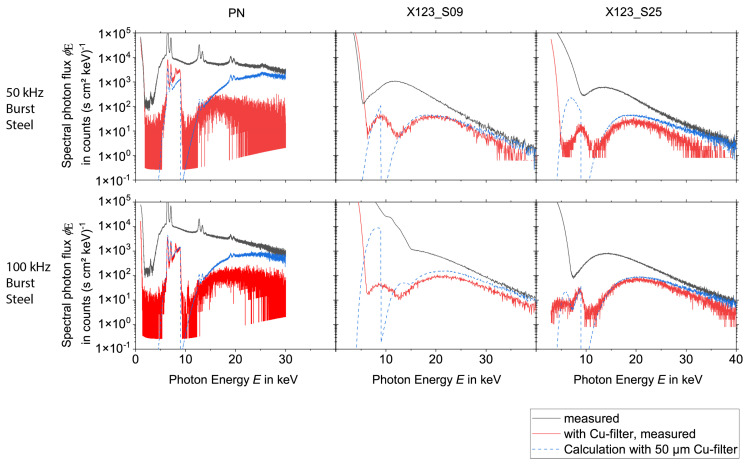
X-ray spectra measured during the processing of steel with burst mode for the three spectrometers PN, X123_S09, and X123_S25, the measurement with and without Cu-filter was compared with the calculation with copper filter of 50 µm thickness. Note the logarithmic scaling of the ordinate axes.

The results for the spectral measurements during the laser processing of tungsten are presented in Figure 4 and Figure 5, while the results during the laser processing of steel are shown in Figure 6 and Figure 7 for all three detectors. Except for experiments no. 1, 3, and 7 (see Table 1) the measurements without copper foil were performed twice for each set of processing parameters and each detector. In the case of two available measurements, the mean value was used for the evaluation. For experiments no. 3 and 7 a single measurement was utilized for the spectrometers X123_S25 and the PN. For experiment no. 1 a single measurement was used for the spectrometers X123_S09 and the PN.

For the PN spectrometer all results, i.e., the comparison of the measurements with copper foil and the corresponding calculations, show a good agreement for X-ray photon energies < 11 keV. However, deviations occur for the measurements with a burst mode for energies > 11 keV, which indicates that pile-up plays a role during the processing with burst mode.

Both X-123 detectors reveal broad spectral peaks with very high intensities for X-ray energies below approximately 9 keV. For the X123_S09, one measurement (100 kHz burst mode, steel) even shows this signal for a photon energy up to 15 keV. It is assumed that these broad peaks are due to EMC issues.

Additionally, also for the X-123 detectors, pile-up can be observed during the laser processing measurements with burst mode. This effect is minimized when the measurement is performed with copper foil. For further evaluation, it follows that the PN spectrometer delivers accurate data (without pile-up) for photon energies < 11 keV. On the other hand, for measurements without burst mode the X-123 spectrometers deliver valid data for photon energies > 13 keV. For energies between 11 and 13 keV, the PN spectrometer data are only valid for processing parameters without burst mode.

As the measurements with burst mode are affected by pile up, the spectra with a copper filter were used. Then the effect of the copper filter was removed by employing Equation (4) and calculating the spectrum without the effects of the copper filter from them.

**Figure 8:**
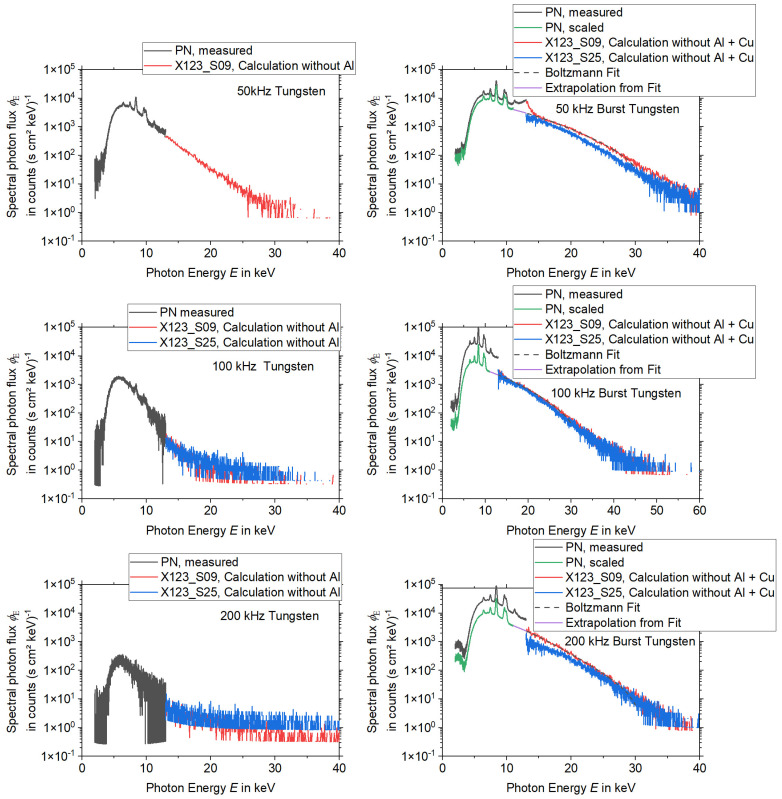
X-ray spectra during the processing of tungsten. Detector type, filter materials, and evaluation constraints are indicated through labels for the different curves. Note the logarithmic scaling of the ordinate axes.

**Figure 9:**
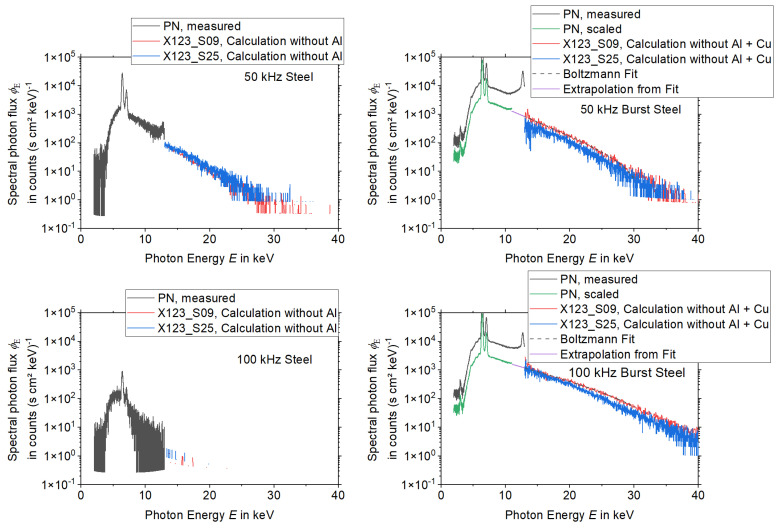
X-ray spectra during the processing of steel. Detector type, filter materials, and evaluation constraints are indicated through labels for the different curves. Note the logarithmic scaling of the ordinate axes.

Due to these findings on the validity of the data in different energy regions, combined spectra with the data from the different detectors were composed for each set of laser processing parameters according to Table 1. The combined spectra for the processing of tungsten can be found in Figure 8, while the spectra for the processing of steel are depicted in Figure 9.

The data from the PN spectrometer were used for photon energies from 2 keV to 13 keV and the data from the X-123 were utilized for energies > 13 keV. As already mentioned, the PN spectrometer data show a slightly increased photon flux for photon energies between 11 and 13 keV upon processing in the burst mode and these data are probably influenced by pile-up effects. This influence is eliminated by a fitting routine which will be explained later in this chapter. For laser processing without burst mode, i.e., in the normal single pulse mode, the X-123 measurements with aluminum foil were used, whereby the spectra before the transmission of the aluminum foil were calculated by using Equation (4). For burst mode laser irradiation, the X-123 measurements with copper and aluminum foil were selected and the influence of the copper and the aluminum filter was considered also by employing Equation (4).

Taking into account the different energy binning and the different sensor sizes for the calculation of the spectral photon flux, the data of the two X-123 spectrometers are in good agreement. For laser processing parameters without burst mode (i.e., in single pulse mode), an almost continuous transition from the PN spectra to the X-123 spectra can be observed. The PN spectra for laser processing parameters with burst mode have an overall significantly higher photon flux than the spectra from the X-123 detectors. The origin of this deviation is unclear. For an estimation of the uncertainty of the spectra, the curves of the X123_S09 were fitted with a Boltzmann function within the photon energy region of 13–30 keV and extrapolated to the energy region between 11 and 13 keV. In the next step, the PN spectra were scaled down in global amplitude until continuous spectra were achieved together with the extrapolation from the Boltzmann fit and the X-123 spectra. As explained before for the burst mode laser processing, the energy region between 2 and 13 keV is associated with some uncertainty. This uncertainty can be quantified by using two combined spectra. On the one hand, the downscaled PN spectrum together with the Boltzmann extrapolation and the X-123 spectrum as a combined spectrum describes the minimum overall flux. The unchanged PN spectrum together with the X-123 spectrum in a combined spectrum characterizes the maximum overall flux.

The comparison of the spectra recorded with and without the laser burst mode reveals a significant increase in the photon flux for the burst mode processing. A detailed analysis of the estimated dose rates from the spectra will be presented later in Section 3.3. An increase in the spectral photon flux can be observed for lower laser repetition rates. This increase can be found for both the processing with and without burst mode. As expected from previous publications, e.g., [4] the spectral photon flux emitted during the processing of tungsten is higher when compared to the processing of steel. This is mainly due to the higher atomic number *Z* of tungsten (*Z* = 74) compared to iron (*Z* = 26) [4]. The maximum energies are limited to about 40 keV. The maximum energy of 40 keV was observed during the laser processing of tungsten and steel with the burst mode with 50 and 100 kHz laser pulse repetition rates. For the processing of tungsten with a higher repetition rate (200 kHz), the maximum X-ray energy is approximately 35 keV. For the laser processing without burst mode, i.e., for single pulse mode, a signal for energies up to 40 keV was measured in the case of the 50 kHz repetition rate. However, with a spectral photon flux of around one count (s cm^2^ keV)^−1^ the signal is very low. Compared to the burst mode, for the processing without burst mode, the spectral photon flux significantly decreases already for lower energies and reaches the minimum spectral photon flux of about one count (s cm^2^ keV)^−1^ for energies between 20 and 30 keV. As a general trend, the maximum X-ray photon energy tends to increase with higher laser pulse energies.

Altogether, the “worst-case” processing scenario of this study is represented by the laser processing of tungsten at a repetition rate of 50 kHz and in burst mode.

### 3.3. Dose Rate Estimations

The X-ray dose rates (*Ḣ*′(0.07), *Ḣ*′(3), and Ḣ*(10)) were calculated from the measured spectra at a distance of 60 cm.

The ambient dose equivalent rate corresponds to the dose equivalent per time at different depths of penetration in the ICRU sphere [28]. Due to the energy-dependent absorption properties of ICRU tissue, three depths of penetration *d_p_* were used. The ambient dose equivalent rates can be calculated from the spectral photon flux *Φ*(*E*) along with the tissue properties via

(5) $$\dot{H}\left(d_{p}\right)=\overset{E=\infty \;}{\underset{E=0}{\int }}\Phi \left(E\right)\frac{E_{\mathrm{Photon}}}{∆E}\;\;\frac{e^{-\mu_{en}^{t}\left(E\right)\;d_{p}}\;\mu_{t}\left(E\right)}{\rho_{}^{t}}dE,$$

where *E* is the photon energy, *E*_Photon_ is the channel energy, ∆*E* is the bin size from the channel (energy channel’s width) and the tissue properties are the X-ray photon energy-dependent energy absorption coefficient $\mu_{en}^{t}$(*E*) of Tissue (Soft ICRU-44) and its density *ρ^t^*. [9]. The data for the attenuation coefficients and density of standard tissue were taken from the NIST database [28]. Equation (5) is a good approximation for low-energy spectra, where the scattering of the X-ray photons can be neglected. Another method to determine the ambient dose equivalent rates would be to employ conversion coefficients from ICRU report 57 [29], for example, used in [30] to determine the spectral ambient dose equivalent rate from the spectral photon fluence and then integrate overall available photon energies. Here, both methods have been compared and due to the low energy of the spectra, no significant deviation has been observed.

**Figure 10:**
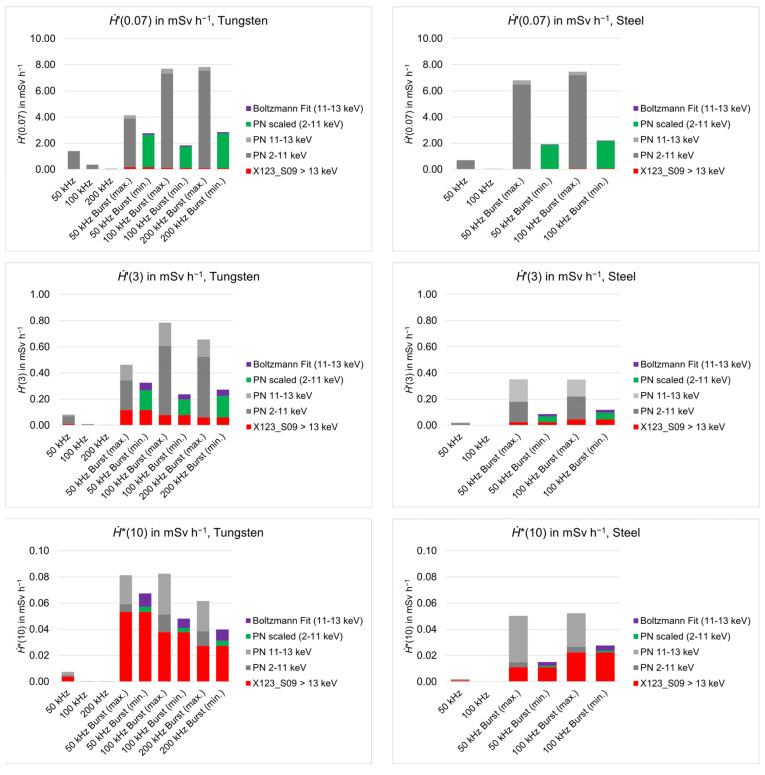
Dose equivalent rate estimations for the quantities *Ḣ*′(0.07), *Ḣ*′(3), and Ḣ*(10), as calculated from the spectra presented in Figure 8 and Figure 9. The results for laser processing of tungsten are summarized in the left column, while the results for laser processing of steel are given in the right column.

The results of the dose equivalent rate calculations for the three quantities *Ḣ*′(0.07), *Ḣ*′(3), Ḣ*(10) are presented in Figure 10. The dose equivalent rates were calculated for the different parts of the combined spectra shown in Figure 8 and Figure 9. Due to the already discussed large uncertainty for the burst mode processing within the photon energy region between 0 and 13 keV, the dose equivalent rates were calculated for the downscaled PN spectra and the Boltzmann extrapolation as well as for the measured PN spectra. This way, a possible maximum and minimum was estimated for the dose equivalent rates. Furthermore, the dose equivalent rate calculations for the different energy regions of the spectra illustrate how the different quantities *Ḣ*′(0.07), *Ḣ*′(3) and Ḣ*(10) are affected by different energy regions of the laser-generated X-ray spectra. Due to the higher penetration depth of X-rays with higher photon energies, the ambient dose equivalent rate Ḣ*(10) depends more on the higher energy region, while the directional dose equivalent rate *Ḣ*′(0.07) depends more on the lower energy region of the spectrum. Therefore, the high uncertainty of the energy region between 0 and 13 keV, which is represented by the data from the PN spectrometer, result in larger differences between the maximum and minimum dose rates and, therewith, in high uncertainties for the quantity *Ḣ*′(0.07). Compared to that, the uncertainty for the ambient dose equivalent rate Ḣ*(10) is smaller. From the “worst-case” spectrum (repetition rate of 50 kHz, burst irradiation mode, tungsten), which was defined due to the highest observed energies, a value of *Ḣ*′(0.07) between 2.8 and 4.2 mSv h^−1^, a value of *Ḣ*′(3) between 0.3 and 0.5 mSv h^−1^ and a value of Ḣ*(10) between 0.07 and 0.08 mSv h^−1^ were estimated. An even higher maximum dose rate *Ḣ*′(0.07) of 7.8 mSv h^−1^ was estimated for the laser processing of tungsten with a repetition rate of 200 kHz in burst mode. However, for these parameters, the deviation between the minimum and maximum dose equivalent rate is significantly larger and, therefore, associated with high uncertainty. If the mean value is calculated from the maximum and minimum dose rate and the uncertainty is calculated by the standard deviation, the maximum dose rate *Ḣ*′(0.07) found in this study is 5.3 ± 3.5 mSv h^−1^ at an operator distance of 60 cm.

The values of *Ḣ*′(3) and Ḣ*(10) are significantly lower, which is due to the lower spectral photon flux within the region of higher energies. Looking at the increase in the dose equivalent rates due to the applied burst irradiation mode compared to the same processing without burst mode, one can see that from *Ḣ*′(0.07) to *Ḣ*′(3) and to Ḣ*(10) the enhancement of the dose equivalent rate increases: for the defined “worst-case” spectrum (repetition rate = 50 kHz, burst mode, tungsten) the value of *Ḣ*′(0.07) is increased by a factor of approximately 2, *Ḣ*′(3) by a factor of approximately 4 and Ḣ*(10) by a factor of approximately 9 when burst mode is applied. This behavior illustrates, that the burst mode laser processing results in a higher spectral photon flux for higher energies and in higher maximum energies. The highest enhancement due to burst mode application could be observed for a laser repetition rate of 200 kHz (tungsten) and a repetition rate of 100 kHz (steel). *Ḣ*′(0.07) was increased by a factor of approximately 60 for these parameters. Although the absolute dose equivalent rates were rather low for these parameters when processing single pulse mode, remarkably high dose rates were measured during the burst mode laser processing.

## 4. Discussion

Within the presented investigation of the X-ray emission during USPL processing, different difficulties during the measurements of X-ray spectra within an USPL machine were encountered. The main problems are associated with pile-up effects, electromagnetic incompatibility, a photon energy-dependent reduced sensitivity of the detector materials and other electronic issues. Minimization of the disturbing influences was achieved by the complementary use of different detector types (CdTe sensor in combination with an SDD) and the application of shielding and reduction in pile-up by using aluminum and copper foils as filters, whereby an evaluation routine that eliminates the absorption effects due to the foils from the measured spectra had to be applied. The radiation protection operational quantities *Ḣ*′(0.07), *Ḣ*′(3), and Ḣ*(10) were calculated from the spectra.

Different combinations for the laser pulse energy (varied between 91 µJ and 371 μJ) and repetition rate (varied between 50 kHz and 200 kHz) were selected for the investigations, whereby the average laser power was kept nearly constant. It was shown that the average laser power cannot be used as the main parameter for an estimation of the dose equivalent rates. The measured dose equivalent rates significantly depend on the combination of the laser pulse repetition rate and the selected pulse energy. Parameter combinations with high laser pulse energy and low repetition rate show significantly higher dose rates than combinations with a high repetition rate and low laser pulse energy although they have almost equivalent average laser power, especially in the case of single pulse operation. Additionally, the burst mode laser operation significantly increases the dose rates although the average laser power remains the same when the burst mode is applied.

The increase in dose equivalent rate during burst mode processing was already described in the literature. A possible explanation for the significant increase in the X-ray dose rate is the interaction between ultrafast laser radiation and the formed ablation cloud or a high-density plasma [20,21].

Metzner et al. [20] investigated the dependence of the number of pulses per burst and the total fluence on the resulting X-ray dose rate by ablating stainless steel (X100CrMoV-8-1-1) with ultrafast laser pulses emitting a wavelength of 1030 nm and a pulse duration of 0.24 ps in the MHz-(MBM), GHz-(GBM), and the BiBurst (BBM) mode in comparison with conventional ultrafast laser radiation in the single-pulse mode (SPM). Compared to the SPM, producing a maximum dose equivalent rate *Ḣ*′(0.07) of approximately 7 × 10^3^ μSv h^−1^, an increase in the X-ray dose rate by more than a factor of 30 was found in the BBM with two and three sub-pulses in the MHz- and two sub-pulses in the GHz-bursts. For these parameters a dose rate of *Ḣ*′(0.07) = 2.5 × 10^5^ µSv h^−1^ was determined. This is within the range between a factor of 2 and 60 which was determined within this study for the increase in the dose equivalent rate *Ḣ*′(0.07) due to burst mode application. Additionally, the absolute values of *Ḣ*′(0.07) are comparable with the results presented here for steel and burst mode taking into account the detector distance of 10 cm in [20] and 60 cm in this work.

Schille et al. [21] observed a significant increase in the dose equivalent rate of the emitted X-rays accompanied by pronounced characteristic X-ray emissions for laser bursts irradiating at a 1.0 MHz burst repetition frequency and corresponding lower peak intensity of the intra-burst pulses. The analysis was performed during the processing of technical-grade AISI 304 stainless steel targets. The maximum X-ray emissions were measured to *Ḣ*′(0.07) = 32.8 ± 3.6 mSv h^−1^ (3rd scan) with the two-pulse burst mode. For burst and bi-burst pulses, the second intra-burst pulse was found to significantly enhance the X-ray emission potentially induced by the laser pulse and plasma interaction. The measured X-ray spectra showed an energy region between 0 and 15 keV, which were measured with a SiLi-based detector. This is significantly lower than the maximum energy of 40 keV, which was observed in this study for burst mode processing of tungsten at a repetition rate of 50 kHz and a laser pulse energy of 371 µJ. Until now, maximum X-ray energies up to 30 keV were reported by Legall et al. [4] during USPL processing of tungsten in single-pulse mode. Additionally, higher dose equivalent rates of *Ḣ*′(0.07) = 163 mSv h^−1^ for the processing of tungsten with a repetition rate of 400 kHz and a peak irradiance of 2.6 × 10^14^ W cm^−2^ were reported in a distance of 42 cm. The maximum X-ray photon energy must be considered if a safety housing is configured for a USPL machine. Legall et al. [12] calculated the equivalent thickness of different shielding materials for X-ray protection up to photon energies of 60 keV in units of iron. Since the here observed maximum energy of 40 keV is lower, these recommendations should still be valid here. The knowledge about the spectral flux of the emitted X-ray photons can also support the work on new testing concepts for USPL machines. Since the actual testing concept, which is based on the “worst-case” processing scenario, is connected with many difficulties [14] a new testing concept that is based on an alternative X-ray source is highly demanded.

To summarize, a “worst-case” spectrum with X-ray photon energies up to 40 keV was found in this study for the machining of tungsten at a repetition rate of 50 kHz and in burst operation mode. Additionally, equivalent dose rates *Ḣ*′(0.07) of the order of 200 mSv h^−1^ were calculated for the processing of tungsten with 200 kHz repetition rate, burst mode and a peak irradiance of 7.3 × 10^13^ W cm^−2^ if a distance between the laser interaction point and detector of 10 cm is assumed without consideration of the absorption in air.

It should be noted here that if the “worst-case” spectrum from USP laser machining is known, it can be simulated by an X-ray tube as a model system for mimicking the USP laser machine. This may lead to a simplified testing concept for USP laser machines in the future, which does not depend on the unstable USP laser-generated plasma as an X-ray source during the testing of the safety housing and is compatible with current standard testing procedures.

## 5. Conclusions

Ultrashort pulse laser processing of tungsten and steel was investigated with respect to the generation of undesired secondary X-rays. Spectral X-ray measurements were performed with complementary CdTe- and SDD-based spectrometers. The radiation protection operational quantities *Ḣ*′(0.07), *Ḣ*′(3), Ḣ*(10) were calculated from the measured X-ray spectra. Regarding the laser processing strategy, the dose equivalent rate of the X-rays increases for lower repetition rates and higher pulse energy if a constant average power is used. The average laser power of ultrashort pulse laser processing alone does not qualify as a suitable parameter for the prediction of X-ray dose rates. Processing with pulse burst mode significantly increases the dose rates and the X-ray photon energies. A maximum dose rate *Ḣ*′(0.07) of 5.3 ± 3.5 mSv h^−1^ at a distance of 60 cm was found for the laser processing of tungsten with a repetition rate of 200 kHz in burst mode and a peak irradiance of 7.3 × 10^13^ W cm^−2^. A maximum X-ray photon energy of about 40 keV was observed for burst mode processing of tungsten with a repetition rate of 50 kHz and a peak irradiance of 3 × 10^14^ W cm^−2^.

## Figures and Tables

**Table 1:** Experimental processing parameters.

Experiment No.	Repetition Rate	Laser Pulse Energy	Average Power	Burst Mode	Peak Irradiance	Material
	in kHz	in µJ	in W		in W cm^−2^	
1	50	371	18.6	No	3.0 × 10^14^	tungsten
2	100	184	18.4	No	1.5 × 10^14^	tungsten
3	200	91	18.2	No	7.3 × 10^13^	tungsten
4	50	371	18.6	Yes	3.0 × 10^14^	tungsten
5	100	184	18.4	Yes	1.5 × 10^14^	tungsten
6	200	91	18.2	Yes	7.3 × 10^13^	tungsten
7	50	371	18.6	No	3.0 × 10^14^	steel
8	100	184	18.4	No	1.5 × 10^14^	steel
9	50	371	18.6	Yes	3.0 × 10^14^	steel
10	100	184	18.4	Yes	1.5 × 10^14^	steel

## Data Availability

The data presented in this study are available on request from the corresponding author.
